# Development and Validation of Prediction Model for Risk Reduction of Metabolic Syndrome by Body Weight Control: A Prospective Population-based Study

**DOI:** 10.1038/s41598-020-67238-5

**Published:** 2020-06-19

**Authors:** Solam Lee, Hunju Lee, Jung Ran Choi, Sang Baek Koh

**Affiliations:** 1 Department of Preventive Medicine, Yonsei University Wonju College of Medicine, Wonju, Republic of Korea; 2 Department of Dermatology, Yonsei University Wonju Severance Christian Hospital, Wonju, Republic of Korea; 3 Institute of Genomic Cohort, Yonsei University Wonju College of Medicine, Wonju, Republic of Korea

**Keywords:** Cardiology, Endocrinology, Health care

## Abstract

Several studies have reported that weight control is of paramount importance in reducing the risk of metabolic syndrome. Nevertheless, this well-known association does not provide any practical information on how much weight loss in a given period would reduce the risk of metabolic syndrome in individuals in a personalized setting. This study aimed to develop and validate a risk prediction model for metabolic syndrome in 2 years, based on an individual’s baseline health status and body weight after 2 years. We recruited 3,447 and 3,874 participants from the Ansan and Anseong cohorts of the Korean Genome and Epidemiology Study, respectively. Among the former, 8636 longitudinal observations of 2,412 participants (70%) and 3,570 of 1,034 (30%) were used for training and internal validation, respectively. Among the latter, all 15,739 observations of 3,874 participants were used for external validation. Compared to logistic regression, Gaussian Naïve Bayes, random forest, and deep neural network, XGBoost showed the highest performance (area under curve of 0.879) and a significantly enhanced calibration of the predictive score with the prevalence rate. The model was ported onto an application to provide the 2-year probability of developing metabolic syndrome by simulating selected target body weights, based on an individual’s baseline health profiles. Further prospective studies are required to determine whether weight-control programs could lead to favorable health outcomes.

## Introduction

Metabolic syndrome is characterized by a cluster of hypertension, dyslipidemia, central obesity, and disturbed glucose control^1^. It is an important risk factor for major adverse cardiovascular events (MACE), a leading cause of death worldwide, such as myocardial infarction, heart failure, and stroke^2–4^. However, the prevalence of metabolic syndrome is expected to continually increase given the rise in overnutrition and sedentary lifestyles, resulting in obesity^5,6^.

However, metabolic syndrome has a reversible nature^1^. Some studies have suggested that the risk of incident MACE could be reduced with an appropriate intervention for metabolic syndrome^7–10^. A recent nationwide study of 10 million persons reported that recovery from metabolic syndrome significantly lowered the risk of MACE, with an incidence rate of 0.85^11^.

By definition, metabolic syndrome represents heterogeneous metabolic statuses in terms of blood pressure, glucose level, and lipid profile. However, weight control is of paramount importance in the overall control of metabolic syndrome^12–15^. Nevertheless, this well-known association does not provide any practical information on how much weight loss in a given period would reduce the risk of metabolic syndrome in individuals. In addition, a single estimate, despite being a major determining factor, may not provide reliable information for risk reduction, given that heterogenous lifestyles and genetic factors across individuals would affect the risk in a different way^16–18^.

We found that a prediction model for reducing the risk of metabolic syndrome could be developed with a large-scale analysis of repeatedly measured data derived from a population-based data source. The aim of this study was to develop and validate a model that predicts 2-year metabolic syndrome based on an individual’s baseline health status and body weight after 2 years.

## Results

### Participants

**Figure 1:**
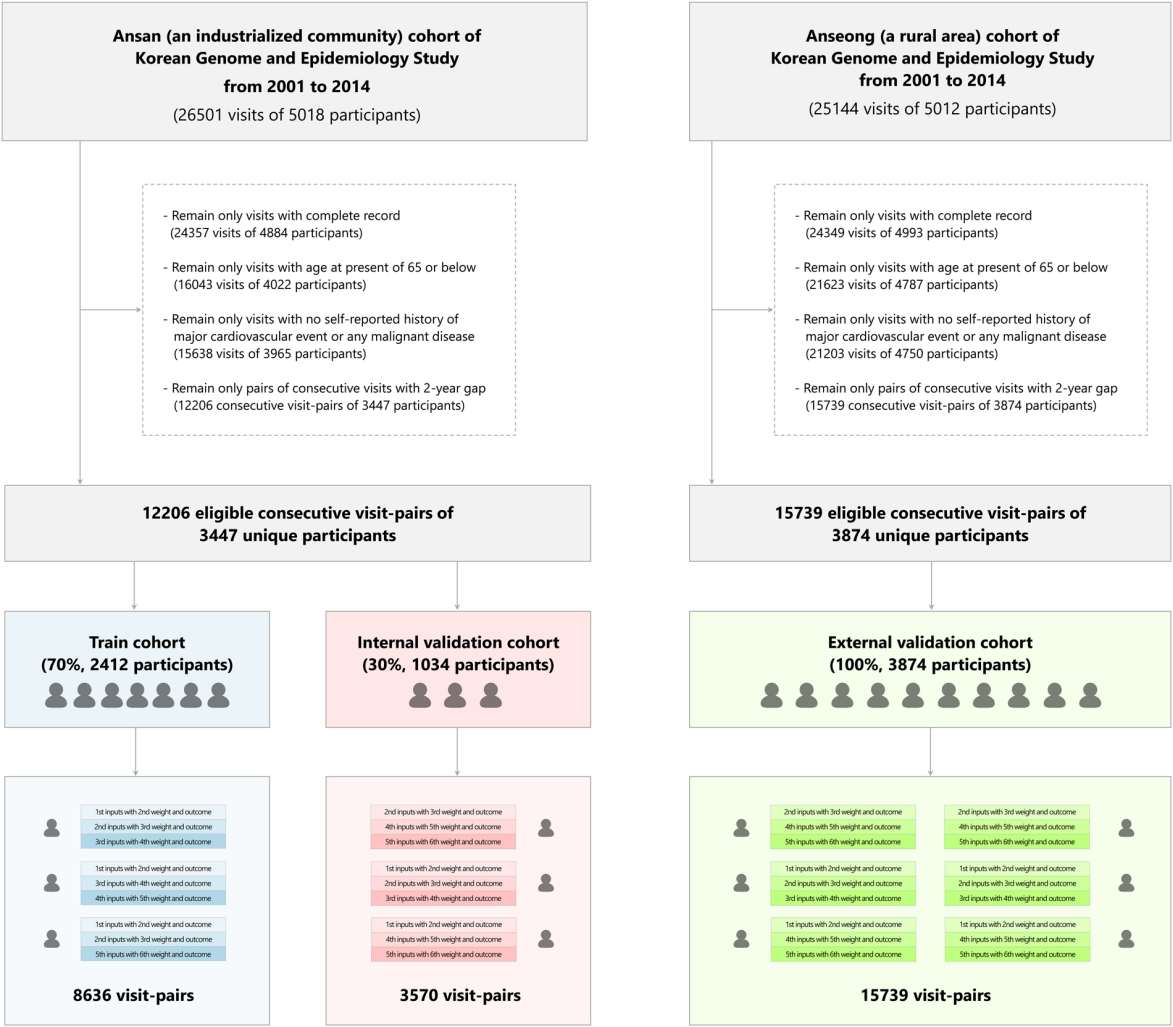
Participant selection flowgram. The community-based Korean Genome and Epidemiology Study (KoGES) was our data source. Among its two sub-cohorts, 12,206 eligible consecutive visit-pairs of 3447 participants were established from the Ansan cohort that represents an industrialized community. Of them, visit-pairs of 2412 participants (70%) were used for training, and of 1034 participants (30%), for internal validation of the model. On the other hand, 15,739 visit-pairs of 3874 participants from the Anseong cohort that represents a rural area were used for external validation of the model.

In total, 12,206 eligible consecutive visit-pairs of 3,447 participants and 15,739 visit-pairs of 3,874 participants were extracted from the Ansan cohort, which represents an industrialized community, and Anseong cohort, which represents a rural area, respectively (Fig. 1)^19^. From the former one, 8,636 visit-pairs of 2,412 participants (70% of 3,447 participants) were used for training, and 3,570 visit-pairs of 1034 participants (30%) were used for internal validation of the models. On the other hand, from the latter one, all 15,739 visit-pairs of 3,874 participants (100%) were used for external validation of the models. The baseline characteristics at the initial visit of the participants are summarized in Table 1.

**Table 1:** Baseline characteristics by study cohort.

Characteristics	Ansan Cohort		Anseong Cohort
	Training Cohort (n = 2412)	Internal Validation Cohort (n = 1035)	External Validation Cohort (n = 3874)
Age, year	53.1 ± 7.5	53.6 ± 7.4	48.4 ± 6.8
**Sex, n (%)**			
Male	1028 (42.6%)	477 (46.1%)	1997 (51.5%)
Female	1384 (57.4%)	558 (53.9%)	1877 (48.5%)
Height, cm	158.9 ± 8.7	159.2 ± 8.4	161.8 ± 8.3
Weight, kg	62.1 ± 9.9	62.5 ± 9.7	64.8 ± 9.9
**Self-reported lifestyle**			
Alcohol intake			
Ever, n (%)	1208 (50.1%)	521 (50.3%)	2229 (57.5%)
Current, n (%)	1053 (43.7%)	439 (42.4%)	2028 (52.3%)
Amount, g/week	8.8 ± 22.1	8.1 ± 19.1	10.7 ± 23.6
Smoking			
Ever, n (%)	885 (36.7%)	396 (38.3%)	1601 (41.3%)
Current, n (%)	585 (24.3%)	269 (26.0%)	882 (22.8%)
Amount, cigarettes	9.7 ± 14.3	10.2 ± 14.5	8.9 ± 12.2
Duration, year	7.2 ± 11.5	7.2 ± 10.8	7.5 ± 11.4
**Self-reported history, n (%)**			
Hypertension	313 (13.0%)	144 (13.9%)	362 (9.3%)
Diabetes mellitus	37 (1.5%)	15 (1.4%)	109 (2.8%)
Dyslipidemia	34 (1.4%)	17 (1.6%)	126 (3.3%)
Gout	119 (4.9%)	54 (5.2%)	142 (3.7%)
**Metabolic syndrome**			
Yes, n (%)	705 (29.2%)	285 (27.5%)	708 (18.3%)
**Component, n (%)**			
Waist circumference	955 (39.6%)	414 (40.0%)	732 (18.9%)
Triglyceride	846 (35.1%)	353 (34.1%)	1360 (35.1%)
High-density lipoprotein	1014 (42.0%)	422 (40.8%)	1445 (37.3%)
Glucose	389 (16.1%)	140 (13.5%)	628 (16.2%)
Blood pressure	1044 (43.3%)	466 (45.0%)	1146 (29.6%)
**No. of components, n (%)**			
0	485 (20.1%)	191 (18.5%)	1099 (28.4%)
1	639 (26.5%)	305 (29.5%)	1186 (30.6%)
2	583 (24.2%)	254 (24.5%)	881 (22.7%)
3	431 (17.9%)	178 (17.2%)	501 (12.9%)
4	220 (9.1%)	87 (8.4%)	175 (4.5%)
5	54 (2.2%)	20 (1.9%)	32 (0.8%)

### Model development and validation

**Figure 2:**
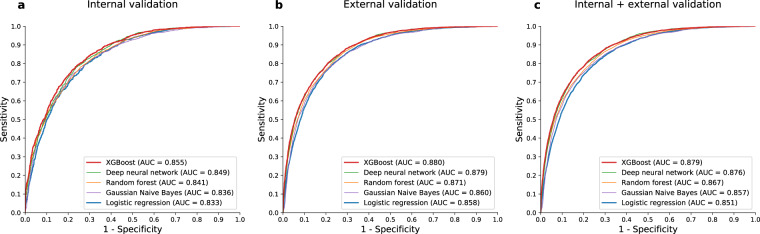
Predictive performances. Receiver operating characteristics curves for machine learning and logistic regression models for (**a**) internal validation, (**b**) external validation, and (**c**) internal + external validation, respectively. Although the difference was not considerable, XGBoost consistently showed the greatest AUC both in internal and validation. Abbreviations: AUC, area under receiver operating characteristics curve.

We fitted several models using logistic regression, Gaussian Naïve Bayes^20^, random forest^21^, XGBoost^22^, and deep neural networks^23^. The results of internal and external validation for the trained models are shown in Fig. 2. The area under receiver operating characteristics curve (AUROC) values of the machine learning-based models are slightly greater than those of the logistic regression model. The performance metrics and confusion matrices at the optimal operating point are summarized in Table 2 and Supplementary Table S1, respectively. The XGBoost-based model was selected as our final model because it consistently showed the best performance both in internal and external validation. The result was consistent in two sensitivity analyses, in which (1) the combined dataset from two regions was used for training and validation of the model (Supplementary Fig. S1) and a (2) 4-year prediction model was established (Supplementary Fig. S2). For comparison of performance, the logistic regression-based model was set as a control to represent a conventional statistical approach in the further analyses.

**Table 2:** Performance metrics at optimal operating point.

		Internal validation (n = 3570)	External validation (n = 15739)	Internal + External (n = 19309)
XGBoost	AUC	0.855	0.880	0.879
	Accuracy, %	77.6	85.6	84.0
	Sensitivity, %	66.1	60.3	62.3
	Specificity, %	84.9	91.0	90.1
	PPV, %	73.6	59.3	63.8
	NPV, %	79.7	91.4	89.5
Deep neural network	AUC	0.855	0.874	0.873
	Accuracy, %	77.0	78.9	78.3
	Sensitivity, %	80.4	79.7	80.7
	Specificity, %	74.8	78.8	77.6
	PPV, %	67.1	44.9	50.2
	NPV, %	85.7	94.7	93.5
Random forest	AUC	0.843	0.868	0.865
	Accuracy, %	76.9	84.5	83.0
	Sensitivity, %	64.4	58.2	60.3
	Specificity, %	84.9	90.1	89.4
	PPV, %	73.1	56.1	61.3
	NPV, %	78.9	90.9	89.0
Gaussian Naïve Bayes	AUC	0.836	0.860	0.857
	Accuracy, %	76.3	83.7	82.3
	Sensitivity, %	67.9	62.9	64.6
	Specificity, %	81.6	88.2	87.2
	PPV, %	70.2	53.7	58.6
	NPV, %	79.9	91.7	89.8
Logistic regression	AUC	0.833	0.859	0.851
	Accuracy, %	75.7	82.8	81.4
	Sensitivity, %	59.2	63.7	62.1
	Specificity, %	86.2	87.0	86.8
	PPV, %	73.2	51.4	56.9
	NPV, %	76.8	91.7	89.1

Abbreviations: AUC, area under curve; PPV, positive predictive value; NPV, negative predictive value.

### Metabolic Syndrome Prediction Index

**Figure 3:**
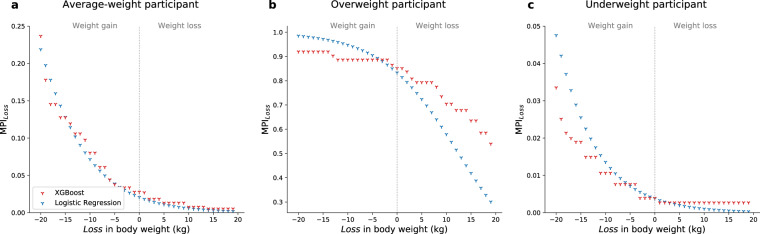
Metabolic syndrome predictive index (MPI) by targeted body weight after 2 years. MPI_*Loss*_ (where *Loss* is the amount of loss in body weight during 2 years) according to the targeted body weight in (**a**) an average-weight participant (BMI of 21.2 kg/m^2^), (**b**) an overweight participant (BMI of 31.2 kg/m^2^), and (**c**) an underweight participant (BMI of 17.6 kg/m^2^), respectively. Unlike logistic regression that did not show any saturation with extensive weight gain in an overweight participant or weight loss in an underweight participant, MPI_*Loss*_ estimated by XGBoost showed a plateau (or inversed plateau) and did not change sensitively in such participants. Abbreviations: MPI, metabolic syndrome predictive index; BMI, body mass index.

The Metabolic Syndrome Prediction Index (MPI_*Loss*_, where *Loss* is the amount of loss in body weight), which represents how likely an individual is to develop metabolic syndrome after 2 years with a target body weight, could be drawn from the model. Figure 3 shows illustrative examples of the application of MPI_*Loss*_ in three differently obese individuals. In a normal-weight participant (body mass index [BMI] of 21.2 kg/m^2^, Fig. 3a), the trends in MPI_*Loss*_ estimated by XGBoost and logistic regression were nearly identical. However, the slopes showed some differences in an overweight participant (BMI of 31.2 kg/m^2^, Fig. 3b) or underweight participant (BMI of 17.6 kg/m^2^, Fig. 3c). Moreover, unlike logistic regression, MPI_*Loss*_ estimated by the XGBoost-based model, did not change further with extensive weight gain or loss in such participants.

**Figure 4:**
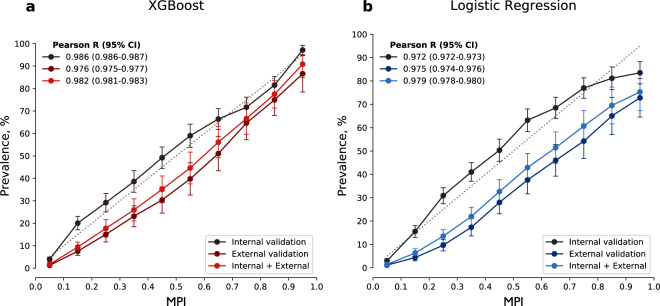
Calibration of metabolic syndrome predictive index and actual prevalence rate after 2 years. The association of the actual prevalence rate of metabolic syndrome after 2 years with MPI_*Loss*_ estimated with (**a**) the XGBoost-based model and (**b**) logistic regression-based model. The linear association was more prominent in the former than in the latter. A relatively low prevalence of metabolic syndrome in the Anseong cohort led to down-deviation of the curves for external validation. Abbreviation: MPI, metabolic syndrome predictive index.

Figure 4 shows the MPI_*Loss*_ estimated from each model and the actual prevalence rate of metabolic syndrome after 2 years in the cohort. Although linear association was observed in both models, the Pearson correlation coefficient of the prevalence rate was significantly greater in MPI_*Loss*_ estimated from XGBoost (Fig. 4a) than that estimated from logistic regression (Fig. 4b) for the internal and external validation cohorts. Therefore, we determined that the XGBoost-based model not only predicts disease status accurately but also yields well-calibrated outputs representing the 2-year probability of developing metabolic syndrome.

The final model was ported onto an in-house web application using the Flask Python library (https://github.com/pallets/flask). When provided with the baseline health profile of a user, the program calculates the probabilities of developing metabolic syndrome after 2 years by running simulations with some selected target body weights (Supplementary Fig. S2). We supplemented a selected package of the final model and simplified the code for its implementation (see Data Availability in Material and Method section).

## Discussion

In this study, we developed and validated a prediction model for metabolic syndrome status after 2 years with an AUROC of over 0.850. In addition, we established an individualized program that can promote weight control by presenting reliable probabilities for having metabolic syndrome.

Metabolic syndrome is a dynamic status^11^. In terms of preventing MACE and its associated mortalities^24,25^, it would be beneficial if an appropriate program for its prevention or treatment could be provided^26–28^. Nevertheless, although several studies have reported that body weight is closely associated with metabolic syndrome, they did not provide a practical goal for weight control and risk reduction in a personalized setting. Setting a realistic goal has significant benefits in health risk reduction^29,30^. Therefore, rather than merely revealing a single statistical estimate of how much the risk can be reduced by a unit of weight reduction, our model can present a realistic probability of risk reduction in an individual with body weight control. In addition, the estimates of our model are based on an individuals’ baseline health profile. The baseline risk and benefits from weight control would be different even among individuals with similar body weight if they have distinct risk factors or components of metabolic syndrome^16–18^.

Our study excluded elderly individuals and patients with a history of MACE or malignant diseases from the analysis for the following reasons: 1) to reduce the potential bias arising from reverse causation because weight loss may be the consequence of severe diseases, 2) metabolic syndrome is one of the important risk factors and strongest predictors of the development of major cardiovascular diseases, and 3) weight reduction is generally not recommended in elderly individuals because being overweight is minimally associated with high mortality in this population^31^. However, the risk of metabolic syndrome is considerably greater in this population than in the normal population. Therefore, the application of these excluding criteria may have caused an overall reduction in the prevalence of metabolic syndrome among the study participants compared to that in the real world. Therefore, the predictive performance of the model could be affected since the excluding criteria can worsen the class imbalance between the participants with and without metabolic syndrome.

Along with logistic regression as a conventional statistical approach, we recruited multiple machine learning-based models. Although the machine learning-based models showed slightly better performance than the logistic regression model, the difference was not considerable (Fig. 2). There may be some reasons behind this observation—i) The data derived from the cohort database were “typical data.” In general, machine learning methods benefit more from “atypical data” (e.g., unprocessed nature language, network, image, and signal data) that can rarely be analyzed by conventional statistical approaches. ii) Among hundreds of variables, a few clinically important variables were selected as input variables^32^. Since this existing knowledge was based on the conventional statistical approach, machine learning could not outperform logistic regression considerably.

The XGBoost-based model had greatest AUROC both in internal and external validation. Unlike logistic regression that assumes log-linearity in all body weights and has equal effect size across individuals, the XGBoost-based model showed a different trend in MPI_*Loss*_, especially in overweight or underweight participants (Fig. 3b,c). Since weight gain or loss would have different effects according to individuals’ baseline health status, this difference could have contributed to some improvement in prediction (e.g., weight loss in underweight individuals would have no or minimal effect in further risk reduction). In addition, MPI_*Loss*_ calculated by XGBoost showed better calibration with the actual prevalence rate. This allowed the model to provide a reliable 2-year probability of developing metabolic syndrome, reflecting real-world data.

In the management of diverse chronic diseases as well as medical intervention by medication and procedures, the importance of lifestyle modification has been greatly emphasized. With the development of digital healthcare, medicine, and therapeutics, the patients’ biometrics are periodically being measured outside the clinic and are increasingly being utilized as adjuvant information in clinical practice. Using our model, patients will be provided the objective effect of weight loss on the risk of metabolic syndrome with considerable reliability, based on each patient’s own cardiovascular risk profile and goal for weight control. Although this study did not validate whether the utilization of our model would achieve a significantly high rate of weight reduction in individuals, as opposed to merely emphasizing the need for weight control in the clinic, our model can be expected to increase compliance considerably.

Our study has some limitations. The study population consisted of participants of a single ethnicity (Asian) and nationality. Further studies are required to determine whether the model can show consistent performance in individuals with different biological and cultural backgrounds. In addition, since the prevalence of metabolic syndrome was low in the study population, a class imbalance in the dataset could have impaired the performance of the models^33^. Nevertheless, this study is advantageous since it established a model that can present a well-calibrated probability that would have a practical meaning for the users, which has rarely been reported. Moreover, this study achieved a considerable generalizability in that the model showed consistent performance not only in internal validation (in an industrialized community) but also in external validation (in a rural area). Further prospective studies are required to determine whether the use of the model-based weight-control program could lead to improved health outcomes.

## Methods

### Data Source and Study Approval

The Korean Genome and Epidemiology Study (KoGES) is a prospective population-based cohort launched in 2001 in South Korea^19^. The community-based KoGES consists of two sub-cohorts: one based on the Ansan region, representing an industrialized community (5012 participants), and the other based on the Anseong region, representing a rural area (5018 participants). The participants of both cohorts have been followed-up every 2 years. The health check-up and measurements of biomarkers are carried out at each visit to identify risk factors for the development of chronic disease such as lifestyle (e.g., alcohol intake, smoking, and exercise), diet profile, and diverse environmental factors. The study was based on data from up to seven repeatedly measured datasets from baseline to 2014 over a 14-year period in the two cohorts. The study was conducted in accordance with the Declaration of Helsinki. Informed written consent was obtained from all participants. Demographic information was collected at the baseline and follow-up examination using a standard questionnaire that was administered during face-to-face interviews. The study was approved by the institutional review board of Yonsei University Wonju Severance Christian Hospital (CR105024).

### Validation of Data Source

The prediction model would be reliable only is the model was trained and validated by the representative data for the general population. We validated the representativeness of our dataset by the additional analyses of the National Health Insurance Service of Korea–National Sample Cohort (NHIS-NSC) as a reference cohort^34^, which includes approximately 1 million individuals, (2% of the total South Korean population). South Korea has a single universal health coverage system providing insurance to over 99% of the South Korean population. Since 2014, the NHIS has made the Bigdata Sharing Service available to researchers; the database includes information recorded since 2002. The health examination results were collected from the general health examination database. This examination is offered (bi)annually to all employees, householders, or any citizen aged 40 years or older.

The data source was validated as follows: (1) by comparing the baseline demographics of the study population in 2001 with those of NHIS-NSC in 2002–2003 (Supplementary Table S2) and (2) by comparing the metabolic syndrome risk profile according to the BMI of the study population in 2009–2010 with those of NHIS-NSC in 2009–2010 (Supplementary Table S3). A separate validation was required because lipid profile (e.g., total cholesterol triglyceride, high- and low-density lipoprotein study) was included in the general health examination since 2009. As a result, we determined that our data source can represent the general population appropriately since the baseline demographics and metabolic syndrome risk profile of the study population did not considerably differ from those of the nationwide cohort database.

### Data Conditioning

We aimed to develop a model that predicts the likelihood of an individual developing metabolic syndrome after 2 years according to weight changes during that period. Therefore, a visit-pair was constructed with the health status at baseline, body weight, and metabolic syndrome status after 2 years (Supplementary Fig. S4). The following baseline characteristics were used as input variables: age, sex, height, weight, alcohol intake (no/current) and amount, smoking status (never/ever/current) and pack-years, systolic and diastolic blood pressure, waist circumferences, fasting glucose level, triglyceride levels, total cholesterol levels, and high-density lipoprotein cholesterol levels. The use of anti-hypertensive, anti-glycemic, or lipid-lowering agents was also reported. The body weight measured after 2 years served as another input variable. The metabolic syndrome status after 2 years was the prediction target of dichotomous classifiers (labeled as 0 for no and 1 for yes). Metabolic syndrome was determined according to the definition described in the next section. Any records with baseline age of 65 years or above and self-reported history of MACE or other malignant diseases were excluded to reduce potential bias caused by reverse causation. Incomplete records with any missing values for the input variables were also excluded. All visit-pair records were established using only the remaining consecutive records collected every 2 years.

### Definition of Metabolic Syndrome

According to the National Cholesterol Education Program Adult Treatment Panel, metabolic syndrome can be confirmed when three or more of the following components are present:^35^ increased waist circumference (≥90 cm for Asian men and ≥80 cm for Asian women), elevated triglyceride level (≥150 mg/dL) or use of a lipid-lowering agent, reduced high-density lipoprotein cholesterol level (≤40 mg/dL for men and ≤50 mg/dL for women), elevated blood pressure (systolic blood pressure ≥130 mmHg or diastolic blood pressure ≥80 mmHg) or use of an antihypertensive agent, and elevated fasting glucose level (≥100 mg/dL) or use of a sugar-lowering agent.

### Dataset, Model Training, and Validation

We used the Ansan cohort among for development and internal validation of the model. On the other hand, the Anseong cohort was used for external validation. To avoid overestimation of the predictive performance, all data partitioning was done on a per-participant basis. For the Ansan cohort, 70% of the unique participants were allocated to the train cohort and 30%, to the internal validation cohort. The cohort assignment was based on the pseudorandom number generator without any stratifying or matching variables. The visit-pairs of the train cohort were then used to determine the optimized parameters to predict the outcome; those of the internal and external validation cohorts were used to evaluate the performance of the trained model in a homogenous and heterogeneous setting, respectively. There was no overlap between the cohorts.

We recruited logistic regression, gaussian Naïve Bayes^20^, random forest^21^, XGBoost^22^, and deep neural network^23^ as potential candidates for developing our model. In the logistic regression, all variables were input at once without any variable-selection algorithm. No significant collinearity among the input variables was detected. The Naïve Bayes classifier^20^ is a probabilistic classifier and among the simplest Bayesian network models. The random forest^21^ and XGBoost^22^ are machine learning algorithms based on a combination of decision trees and a gradient-boosting framework, respectively. Two algorithms were optimized with the grid searches^36^ and trained with 500 epochs with 5-fold cross validation. Deep neural network^23^ is a method in which complex hierarchical representations are learned with multiple levels of abstraction. A fully connected multilayer perceptron was recruited. There were two hidden layers with 200 nodes. Each layer included the rectified linear unit function for non-linear activation. The loss function was binary cross entropy and the optimizer was Adam with a learning rate of 5 × 10^−3^. In all four algorithms, the output was transformed to have a numeric value between 0 and 1, representing the confidence score for metabolic syndrome after 2 years, referred to as MPI_*Loss*_.

### Statistical Analysis

The baseline characteristics at study entry were summarized using the mean with standard deviation and frequency with percentage, as appropriate. The AUROC was the primary measure of model performance. The optimal operating point was determined at the point at which the Youden index was maximized^37^. The accuracy, sensitivity, specificity, positive predictive value, and negative predictive value were also calculated.

1 $$Youden\,index=Sensitivity\left(\frac{True\,positives}{True\,positives+False\,negatives}\right)+Specificity\left(\frac{True\,negatives}{True\,negatives+False\,positives}\right)-1$$

2 $$Accuracy=\frac{True\,positive+True\,negative}{True\,positives+True\,negatives+False\,positives+False\,negatives}\times 100\,( \% )$$

A 2-tailed *P* value of <0.05 was used to determine the statistical significance. All statistical analyses and the development and validation of the model were carried out with Python version 3.7.0 with pandas (http://pandas.pydata.org) and the scikit-learn (http://scikit-learn.org) library.

### Sensitivity Analysis

Two sensitivity analyses were conducted to ensure the robustness of our model. 1) Rather than performing external validation with a separate dataset, the combined dataset from the two regional cohorts was used for model training and validation. This was done to prevent over-fitting due to under-presentation of certain populations among a single regional cohort. 2) The study was repeated with visit-pairs established with consecutive records from every 4 years, instead of 2 years. Therefore, this model predicted the metabolic syndrome status after 4 years. However, the weight-control program is practically feasible when it can present a short-term goal. Therefore, the 2-year prediction model was retained as our main model.

## Supplementary information

Supplementary information.

## Data Availability

All Python codes used for data preprocessing, model development, and validation are published in our public repository [10.17632/j4x3c8v8bh.1]. In addition, the parameters of the final model and simplified code for demonstration can also be accessed. The data that support the findings of this study are available from the National Research Institute of Health of South Korea, but restrictions apply to the availability of these data, which were used under license for the current study and are not publicly available. Data are however available from the authors upon reasonable request and with permission of the National Research Institute of Health of South Korea. Further information is available at the KoGES website [https://www.cdc.go.kr/menu.es?mid=a50401010100].
