# Supplementary material for: Development and Validation of Prediction Model for Risk Reduction of Metabolic Syndrome by Body Weight Control: A Prospective Population-based Study
Source: Sci Rep. 2020 Jun 19;10:10006. doi: 10.1038/s41598-020-67238-5 (PMC7305222; doi:10.1038/s41598-020-67238-5)
Supplement: Supplementary file 1 — Supplementary information. [file 41598_2020_67238_MOESM1_ESM.docx]

**Supplementary Materials for Article**

**Development and Validation of Prediction Model for Risk Reduction of Metabolic Syndrome by Body Weight Control: A Prospective Population-based Study**

Solam Lee, MD^1,2^; Hunju Lee, MD^1^; Jung Ran Choi, PhD^3^; and Sang Baek Koh, MD, PhD^1^

^1^Department of Preventive Medicine, Yonsei University Wonju College of Medicine, Wonju, Republic of Korea

^2^Department of Dermatology, Yonsei University Wonju Severance Christian Hospital, Wonju, Republic of Korea

^3^Institute of Genomic Cohort, Yonsei University Wonju College of Medicine, Wonju, Republic of Korea

**Corresponding author:** Sang Baek Koh, MD, PhD

Department of Preventive Medicine, Yonsei University Wonju College of Medicine,
20 Ilsan-ro, Wonju, Gangwon-do 26426, Korea

Tel: +82-33-741-0345 / Fax: +82-33-747-0409 / E-mail: kohhj@yonsei.ac.kr

**Date of revision:** May 27, 2020

**Contents**

**Supplementary Figure S1.** Sensitivity analysis by combining two regional cohorts

**Supplementary Figure S2.** Sensitivity analysis by 4-year risk prediction

**Supplementary Figure S3.** An example of application of predictive model as individualized weight-loss program

**Supplementary Figure S4.** An example of derivation of 2-year-gap consecutive visit-pairs in one participant

**Supplementary Table S1.** Confusion matrices at optimal operating point

**Supplementary Table S2.** Demographics of study population and reference cohorts (2001-2002)

**Supplementary Table S3.** Metabolic syndrome profile according to body mass index in reference cohort and study cohort

**Supplementary Figure S1. Sensitivity analysis by combining two regional cohorts**

**
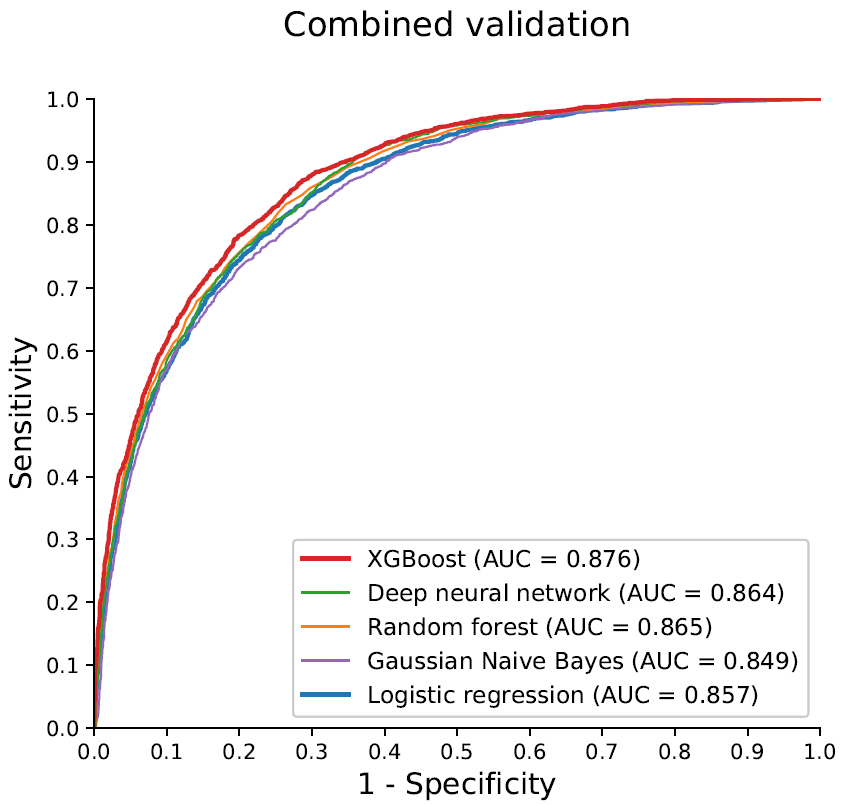
**

**Supplementary Figure S2. Sensitivity analysis by 4-year risk prediction**

**
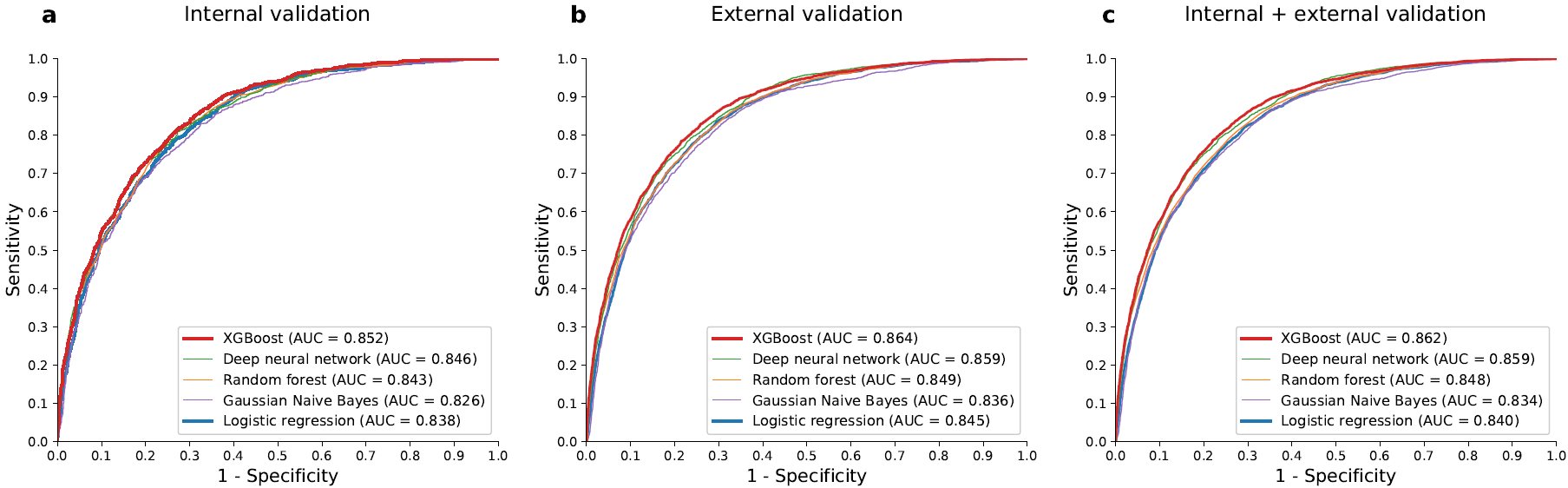
**

**Supplementary Figure S3. An example of application of predictive model as individualized weight-loss program**

**
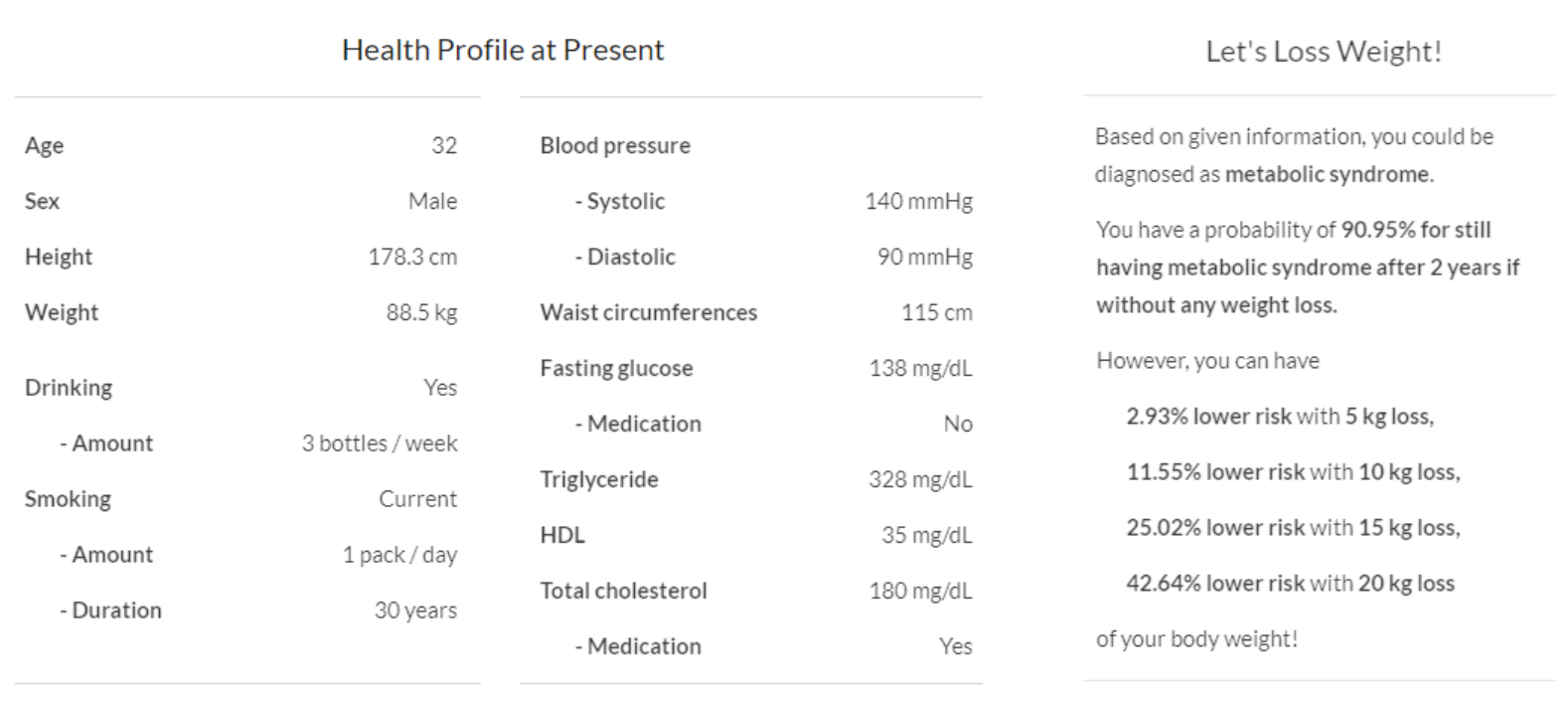
**

**Supplementary Figure S4. An example of derivation of 2-year-gap consecutive visit-pairs in one participant**

**
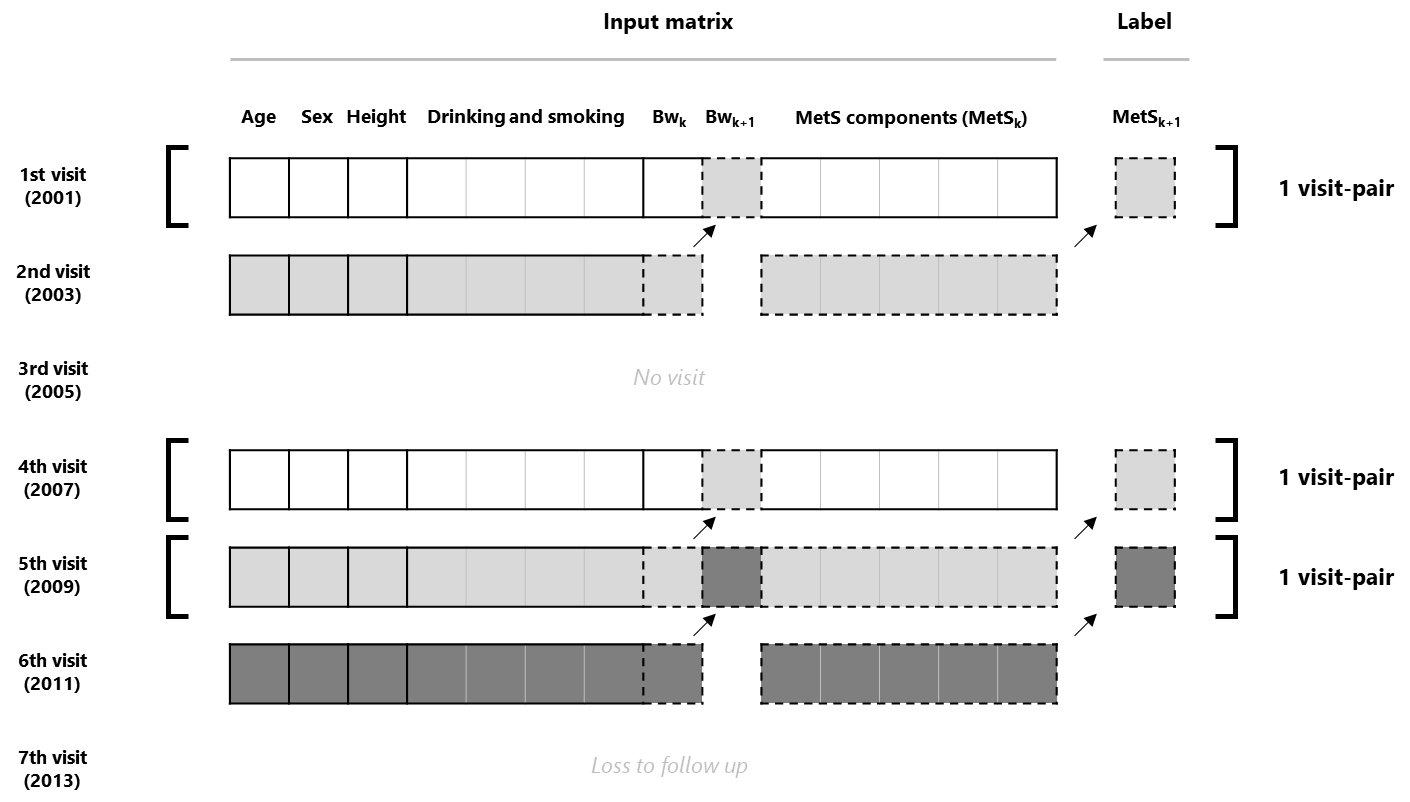
**

**Supplementary Table S1. Confusion matrices at optimal operating point**

**XGBoost**

|  | **Internal validation** | | **External validation** | | **Internal + External** | |
| --- | --- | --- | --- | --- | --- | --- |
|  | **P-MetS(+)** | **P-MetS(+)** | **P-MetS(+)** | **P-MetS(+)** | **P-MetS(-)** | **P-MetS(-)** |
| **O-MetS(+)** | 1938 (51.8) | 345 (9.2) | 11779 (74.8) | 1158 (7.4) | 13212 (67.8) | 2008 (10.3) |
| **O-MetS(-)** | 493 (13.2) | 963 (25.8) | 1113 (7.1) | 1689 (10.7) | 1607 (8.3) | 2651 (13.6) |

Abbreviations: P-MetS, predicted metabolic syndrome; O-MetS, observed metabolic syndrome

**Deep neural network**

|  | **Internal validation** | | **External validation** | | **Internal + External** | |
| --- | --- | --- | --- | --- | --- | --- |
|  | **P-MetS(+)** | **P-MetS(+)** | **P-MetS(+)** | **P-MetS(+)** | **P-MetS(-)** | **P-MetS(-)** |
| **O-MetS(+)** | 1743 (46.6) | 540 (14.4) | 10198 (64.8) | 2739 (17.4) | 11916 (61.2) | 3304 (17.0) |
| **O-MetS(-)** | 321 (8.6) | 1135 (30.4) | 557 (3.5) | 2245 (14.3) | 835 (4.3) | 3423 (17.6) |

**Random forest**

|  | **Internal validation** | | **External validation** | | **Internal + External** | |
| --- | --- | --- | --- | --- | --- | --- |
|  | **P-MetS(+)** | **P-MetS(+)** | **P-MetS(+)** | **P-MetS(+)** | **P-MetS(-)** | **P-MetS(-)** |
| **O-MetS(+)** | 1926 (51.5) | 357 (9.5) | 11761 (74.7) | 1176 (7.5) | 13687 (70.3) | 1533 (7.9) |
| **O-MetS(-)** | 539 (14.4) | 917 (24.5) | 1185 (7.5) | 1617 (10.3) | 1724 (8.9) | 2534 (13.0) |

**Gaussian Naïve Bayes**

|  | **Internal validation** | | **External validation** | | **Internal + External** | |
| --- | --- | --- | --- | --- | --- | --- |
|  | **P-MetS(+)** | **P-MetS(+)** | **P-MetS(+)** | **P-MetS(+)** | **P-MetS(-)** | **P-MetS(-)** |
| **O-MetS(+)** | 1863 (49.8) | 420 (11.2) | 11414 (72.5) | 1523 (9.7) | 13277 (68.2) | 1943 (10.0) |
| **O-MetS(-)** | 468 (12.5) | 988 (26.4) | 1039 (6.6) | 1763 (11.2) | 1507 (7.7) | 2751 (14.1) |

**Logistic regression**

|  | **Internal validation** | | **External validation** | | **Internal + External** | |
| --- | --- | --- | --- | --- | --- | --- |
|  | **P-MetS(+)** | **P-MetS(+)** | **P-MetS(+)** | **P-MetS(+)** | **P-MetS(-)** | **P-MetS(-)** |
| **O-MetS(+)** | 1966 (52.6) | 317 (8.5) | 11246 (71.5) | 1691 (10.7) | 13212 (67.8) | 2008 (10.3) |
| **O-MetS(-)** | 594 (15.9) | 862 (23.1) | 1013 (6.4) | 1789 (11.4) | 1607 (8.3) | 2651 (13.6) |

**Supplementary Table S2. Baseline demographics of study reference cohort and study cohort**

|  |  | **Reference cohort** | | **Study cohort** | | | | | |
| --- | --- | --- | --- | --- | --- | --- | --- | --- | --- |
|  |  | **NHIS-NSC (2002-2003)** | | **Ansan + Anseong (2001)** | | **Ansan (2001)** | | **Anseong (2001)** | |
|  |  | **Male**  **(n = 63288)** | **Female**  **(n = 40937)** | **Male**  **(n = 4165)** | **Female**  **(n = 4552)** | **Male**  **(n = 1770)** | **Female**  **(n = 2228)** | **Male**  **(n = 2895)** | **Female**  **(n = 2324)** |
| **Age (year),**  **n (%)** | **40 – 44** | 15865 (25.1) | 4298 (10.5) | 1288 (30.9) | 1356 (29.8) | 311 (17.6) | 437 (19.6) | 977 (40.8) | 919 (39.5) |
|  | **45 – 49** | 17564 (27.8) | 12843 (31.4) | 1066 (25.6) | 1001 (22.0) | 387 (21.9) | 430 (19.3) | 679 (28.4) | 571 (24.6) |
|  | **50 – 54** | 12656 (20.0) | 9079 (22.2) | 631 (15.2) | 684 (15.0) | 320 (18.1) | 358 (16.1) | 311 (13.0) | 326 (14.0) |
|  | **55 – 59** | 10309 (16.3) | 9174 (22.4) | 603 (14.5) | 699 (15.4) | 344 (19.4) | 443 (19.9) | 259 (10.8) | 256 (11.0) |
|  | **60 – 65** | 6894 (10.9) | 5543 (13.5) | 577 (13.9) | 812 (17.8) | 408 (23.1) | 560 (25.1) | 169 (7.1) | 252 (10.8) |
| **Body mass index (kg/m^2^),**  **n (%)** | **< 18** | 704 (1.11) | 599 (1.5) | 36 (0.9) | 32 (0.7) | 27 (1.5) | 18 (0.8) | 9 (0.4) | 14 (0.6) |
|  | **18.0 – 20.9** | 7539 (11.9) | 7318 (17.9) | 487 (11.7) | 420 (9.2) | 281 (15.9) | 196 (8.8) | 206 (8.6) | 224 (9.6) |
|  | **21.0 – 23.9** | 22418 (35.4) | 16031 (39.2) | 1345 (32.3) | 1412 (31.0) | 587 (33.2) | 635 (28.5) | 758 (31.6) | 777 (33.4) |
|  | **24.0 – 26.9** | 23414 (37.0) | 11660 (28.5) | 1565 (37.6) | 1607 (35.3) | 601 (34.0) | 795 (35.7) | 964 (40.3) | 812 (34.9) |
|  | **27.0 – 29.9** | 7640 (12.1) | 4091 (10.0) | 603 (14.5) | 750 (16.5) | 218 (12.3) | 394 (17.7) | 385 (16.1) | 356 (15.3) |
|  | **> 30** | 1573 (2.5) | 1238 (3.0) | 129 (3.1) | 331 (7.3) | 56 (3.2) | 190 (8.5) | 73 (3.0) | 141 (6.1) |

Abbreviations: NHIS-NSC, National Health Insurance Database-National Sample Cohort

**Supplementary Table S3. Metabolic syndrome profile according to body mass index in reference cohort and study cohort**

|  | **Reference cohort** | | | | | | **Study cohort (4th follow-up)** | | | | | |
| --- | --- | --- | --- | --- | --- | --- | --- | --- | --- | --- | --- | --- |
|  | **NHIS-NSC (2009 – 2010) (n = 233969)** | | | | | | **Ansan + Anseong (2009 – 2010) (n = 4496)** | | | | | |
|  | **< 18.0** | **18.0 – 20.9** | **21.0 – 23.9** | **24.0 – 26.9** | **27.0 – 29.9** | **> 30.0** | **< 18.0** | **18.0 – 20.9** | **21.0 – 23.9** | **24.0 – 26.9** | **27.0 – 29.9** | **> 30.0** |
| **Metabolic syndrome, n (%)** | 95 (2.8) | 35034 (4.5) | 9556 (11.1) | 19823 (27.2) | 13657 (51.2) | 5482 (68.1) | 0  (0.0) | 22 (5.5) | 267 (16.9) | 613 (37.6) | 396 (60.2) | 151 (74.4) |
| **Component, n (%)** |  |  |  |  |  |  |  |  |  |  |  |  |
| **Waist circumference** | 24 (0.7) | 112 (0.3) | 2266 (2.6) | 15794 (21.7) | 17360 (65.1) | 7422 (92.1) | 0  (0.0) | 5 (1.3) | 135 (8.6) | 569 (34.9) | 476 (72.3) | 196 (96.6) |
| **Triglyceride** | 338 (9.9) | 4922 (13.4) | 22531 (26.1) | 30135 (41.4) | 13744 (51.6) | 4373 (54.3) | 2 (6.9) | 60 (15.0) | 421 (26.7) | 666 (40.9) | 305 (46.4) | 95 (46.8) |
| **High density lipoprotein** | 418 (12.3) | 6251 (17.0) | 19755 (22.9) | 21016 (28.9) | 9049 (34.0) | 3075 (38.2) | 13 (44.8) | 155 (38.8) | 837 (53.1) | 997 (61.2) | 466 (70.8) | 151 (74.4) |
| **Glucose** | 611 (17.9) | 7827 (21.3) | 24211 (28.1) | 26888 (36.9) | 11685 (43.84) | 4024 (50.0) | 5 (17.2) | 68 (17.0) | 467 (29.6) | 638 (39.1) | 327 (49.7) | 116 (57.1) |
| **Blood pressure** | 623 (18.3) | 8292 (22.6) | 29099 (33.7) | 34553 (47.4) | 15660 (58.8) | 5583 (69.3) | 4 (13.8) | 77 (19.3) | 406 (25.7) | 541 (33.2) | 270 (41.0) | 95 (46.8) |
| **No. of components, n (%)** |  |  |  |  |  |  |  |  |  |  |  |  |
| **0** | 1950 (57.2) | 17862 (48.7) | 28235 (46.0) | 11971 (16.4) | 1342 (5.0) | 65 (0.8) | 11 (37.9) | 155 (38.8) | 338 (21.4) | 169 (10.4) | 14 (2.1) | 2 (1.0) |
| **1** | 1024 (30.1) | 12215 (33.3) | 29952 (34.7) | 20652 (28.4) | 4422 (16.6) | 703 (8.7) | 12 (41.4) | 148 (37.1) | 551 (34.9) | 400 (24.5) | 95 (14.4) | 8 (3.9) |
| **2** | 339 (9.9) | 4957 (13.5) | 18556 (21.5) | 20411 (28.0) | 7232 (27.1) | 1805 (22.4) | 6 (20.7) | 74 (18.5) | 421 (26.7) | 448 (27.5) | 153 (23.3) | 42 (20.7) |
| **3** | 73 (2.1) | 1363 (3.7) | 7534 (8.7) | 13373 (18.4) | 7484 (28.1) | 2571 (31.9) | 0 (0.0) | 19 (4.8) | 204 (12.9) | 389 (23.9) | 197 (29.9) | 67 (33.0) |
| **4** | 21 (0.6) | 294 (0.8) | 1914 (2.2) | 5457 (7.5) | 4705 (17.7) | 2104 (26.1) | 0 (0.0) | 3 (0.8) | 54 (3.4) | 172 (10.6) | 143 (21.7) | 60 (29.6) |
| **5** | 1 (0.0) | 2 (0.0) | 108 (0.1) | 993 (1.4) | 1468 (5.5) | 807 (10.0) | 0 (0.0) | 0 (0.0) | 9 (0.6) | 52 (3.2) | 56 (8.5) | 24 (11.8) |
|  | **Study cohort (4th follow-up)** | | | | | | | | | | | |
|  | **Ansan (2009 – 2010)** | | | | | | **Anseong (2009 – 2010)** | | | | | |
|  | **< 18.0** | **18.0 – 20.9** | **21.0 – 23.9** | **24.0 – 26.9** | **27.0 – 29.9** | **> 30.0** | **< 18.0** | **18.0 – 20.9** | **21.0 – 23.9** | **24.0 – 26.9** | **27.0 – 29.9** | **> 30.0** |
| **Metabolic syndrome, n (%)** | 0 (0.0) | 14 (8.4) | 157 (25.5) | 362 (58.2) | 203 (74.6) | 74 (74.7) | 0 (0.0) | 8 (3.4) | 110 (11.4) | 251 (24.9) | 193 (50.0) | 77 (74.0) |
| **Component, n (%)** |  |  |  |  |  |  |  |  |  |  |  |  |
| **Waist circumference** | 0 (0.0) | 4 (2.4) | 127 (20.6) | 444 (71.4) | 263 (96.7) | 99 (100.0) | 0 (0.0) | 1 (0.4) | 8 (0.8) | 125 (12.4) | 213 (55.2) | 97 (93.3) |
| **Triglyceride** | 2 (12.5) | 27 (16.3) | 166 (26.9) | 250 (40.2) | 125 (46.0) | 37 (37.4) | 0 (0.0) | 33 (14.2) | 255 (26.5) | 416 (41.3) | 180 (46.6) | 58 (55.8) |
| **High-density lipoprotein** | 9 (56.2) | 82 (49.4) | 355 (57.6) | 427 (68.6) | 213 (78.3) | 77 (77.8) | 4 (30.8) | 73 (31.3) | 482 (50.2) | 570 (56.5) | 253 (65.5) | 74 (71.2) |
| **Glucose** | 4 (25.0) | 37 (22.3) | 219 (35.6) | 295 (47.4) | 138 (50.7) | 56 (56.6) | 1 (7.7) | 31 (13.3) | 248 (25.8) | 343 (34.0) | 189 (49.0) | 60 (57.7) |
| **Blood pressure** | 3 (18.8) | 49 (29.5) | 201 (32.6) | 272 (43.7) | 148 (54.4) | 57 (57.6) | 1 (7.7) | 28 (12.0) | 205 (21.3) | 269 (26.7) | 122 (31.6) | 38 (36.5) |
| **No. of components, n (%)** |  |  |  |  |  |  |  |  |  |  |  |  |
| **0** | 3 (18.8) | 41 (24.7) | 92 (14.9) | 15 (2.4) | 0 (0.0) | 0 (0.0) | 8 (61.5) | 114 (48.9) | 246 (25.6) | 154 (15.3) | 14 (3.6) | 2 (1.9) |
| **1** | 8 (50.0) | 67 (40.4) | 192 (31.2) | 96 (15.4) | 17 (6.2) | 3 (3.0) | 4 (30.8) | 81 (34.8) | 359 (37.4) | 304 (30.2) | 78 (20.2) | 5 (4.8) |
| **2** | 5 (31.2) | 44 (26.5) | 175 (28.4) | 149 (24.0) | 52 (19.1) | 22 (22.2) | 1 (7.7) | 30 (12.9) | 246 (25.6) | 299 (29.7) | 101 (26.2) | 20 (19.2) |
| **3** | 0 (0.0) | 12 (7.2) | 111 (18.0) | 199 (32.0) | 84 (30.9) | 33 (33.3) | 0 (0.0) | 7 (3.0) | 93 (9.7) | 190 (18.8) | 113 (29.3) | 34 (32.7) |
| **4** | 0 (0.0) | 2 (1.2) | 37 (6.0) | 118 (19.0) | 81 (29.8) | 25 (25.3) | 0 (0.0) | 1 (0.4) | 17 (1.8) | 54 (5.4) | 62 (16.1) | 35 (33.7) |
| **5** | 0 (0.0) | 0 (0.0) | 9 (1.5) | 45 (7.2) | 38 (14.0) | 16 (16.2) | 0 (0.0) | 0 (0.0) | 0 (0.0) | 7 (0.7) | 18 (4.7) | 8 (7.7) |

Abbreviations: NHIS-NSC, National Health Insurance Service-National Sample Cohort
